# Case Report: *De novo* variant of the *NUS1* gene associated with developmental delay and autism spectrum disorders in a Chinese family

**DOI:** 10.3389/fped.2025.1557103

**Published:** 2025-05-14

**Authors:** Feng Ding, Junlin Pan, Shuhua Ji, Yan Zhang, Jinwei Hou, Na Shi, Haiping Liu

**Affiliations:** Department of Reproductive Medicine, The 960th Hospital of the People's Liberation Army (PLA) Joint Logistics Support Force, Jinan, China

**Keywords:** *NUS1*, developmental delay, autism spectrum disorders, case report, whole-exome sequencing

## Abstract

**Background:**

Nuclear undecaprenyl pyrophosphate synthase 1 (*NUS1*) has been implicated in the pathogenesis of neurodevelopmental disorders, including Parkinson's disease, seizures, intellectual disability, dystonia, and congenital disorder of glycosylation. To this day, there have been limited studies and reports on the *NUS1* gene.

**Methods:**

We described the case of an 8-year-old Chinese boy exhibiting developmental delay, intellectual disability, and autism spectrum disorder (ASD). To elucidate the genetic etiology, whole-exome sequencing was performed on the proband. A candidate variant was subsequently validated by Sanger sequencing in the proband and his unaffected parents.

**Results:**

Whole-exome sequencing analysis discovered a novel heterozygous variant (c.279del, p.L94Wfs*11) on exon 1 of *NUS1* (NM_138459.5), leading to premature termination of protein translation (p.L94Wfs*11). Sanger sequencing failed to identify the candidate variant in his unaffected parents. Following the updated American College of Medical Genetics and Genomics guidelines, the c.279del variant was classified as pathogenic (PVS1+PM6+PM2_Supporting). Based on the clinical phenotype of the proband, he was diagnosed with autosomal dominant intellectual developmental disorder-55 with seizures (MRD55) and ASD.

**Conclusions:**

This study expands the phenotype and mutation spectrum of the *NUS1* gene, which contributes to the diagnosis of related disorders. Furthermore, the identification of the genetic basis of the proband and confirmation of the corresponding loci of his family members will facilitate the genetic counseling of the proband's parents regarding reproduction.

## 1 Introduction

Autosomal dominant intellectual developmental disorder-55 with seizures (MRD55; OMIM#617831) is a rare hereditary disease characterized by delayed developmental delay, intellectual disability, motor and language impairment, ataxic gait, fine motor dysfunction, tremor, and diverse seizure phenotypes. Nuclear undecaprenyl pyrophosphate synthase 1 (*NUS1*) has been implicated in the pathogenetic progression of MRD55 (1–3). The *NUS1* gene encodes the Nogo-B receptor (NgBR) protein, a conserved subunit of cis-prenyltransferase (cis-PTase) that facilitates dolichol synthesis (4, 5). However, reported cases of NUS1-related disorders remain limited. Based on the Human Gene Mutation Database, a total of 51 likely pathogenic or pathogenic mutations of *NUS1* have been found, of which 20 mutations are associated with Parkinson's disease, 7 mutations are associated with congenital disorder of glycosylation, 2 mutations are associated with dystonia, and the remaining 22 mutations are mainly related to the symptoms of MRD55, such as intellectual disability, developmental delay, and seizures. Further studies are required to better elucidate the correlation between MRD55 and pathogenic variants of the *NUS1* gene.

With the widespread clinical application of second-generation sequencing, whole-exome sequencing has become a routine diagnostic approach for identifying the etiology in children with intellectual disability, developmental delay, and seizures (6–8). This article describes an 8-year-old boy with intellectual disability, delayed speech, delayed movement, and suspected autism spectrum disorder (ASD). He was diagnosed with MRD55 with a rare *de novo NUS1* variant complicated with ASD. Our findings expanded the understanding of *NUS1*-related disorders and elucidated the possible etiology of the case, which provided a reliable genetic basis for clinical diagnosis and genetic counseling.

## 2 Materials and methods

### 2.1 Clinical data

The proband, an 8-year-old boy, was the first child born to healthy non-consanguineous parents. No abnormalities were found in the proband during the fetal and neonatal periods. The proband was delivered via cesarean section at 40 weeks of gestation with a birth weight of 3,500 g. After birth, the proband was able to hold his head up at 3 months, sit at 6 months, crawl at 8 months, stand at 12 months, walk at 16 months, say single words at 24 months, and speak simple sentences at 30 months. The proband's mother reported that the child had experienced two episodes of fever-induced seizures at the age of 3 years. At the age of 6, the proband presented with intellectual disability, delayed speech, delayed movement, stereotyped behavior, shaking of the body, and applauding when happy. There were no obvious abnormalities found in the patient's brain magnetic resonance imaging and electroencephalogram. A neuropsychological assessment using the Wechsler Intelligence Scale demonstrated significantly impaired cognitive functioning, with a full-scale IQ score of 58. The Autism Behavior Checklist score was 58, and the Childhood Autism Rating Scale score was 36. The autism diagnostic observation schedule (second edition) was performed and indicated a moderate risk of autism disorder. The proband was diagnosed with developmental delay and suspected ASD by the local hospital. The patient is currently showing notable improvements in language abilities, motor function, and autistic symptoms while undergoing systematic rehabilitation training, for which continued follow-up monitoring will be maintained.

To elucidate the genetic etiology in this family and prevent the recurrence of similarly affected offspring, the proband's parents opted for comprehensive genetic testing. Whole-exome sequencing was initially performed on the proband, followed by Sanger sequencing validation of the identified variant in both the proband and his parents for segregation analysis. The parents of the proband signed written informed consent, and this study was authorized by the Ethics Committee of the 960th Hospital of the PLA Joint Logistics Support Force.

### 2.2 Whole-exome sequencing and analysis

The extraction of the proband's genomic DNA from peripheral blood was performed using the QIAamp DNA Blood Kit (QIAGEN) following the standard protocols. Based on the Illumina standard protocol, DNA libraries were established. Exome capture was conducted using IDT xGen Exome Research Panel v1.0 and sequencing with the Illumina NovaSeq6000 platform (Illumina Inc., San Diego, CA, USA). Subsequently, the reads were aligned to the GRCh37/hg19 human reference genome through Burrows–Wheeler Aligner software. The GATK Toolkit was used to obtain variant calling, while ANNOVAR software was used to acquire variant annotation. Furthermore, high-frequency variants were filtered out according to databases including database of Short Genetic Variations (dbSNP), Exome Sequencing Project (ESP), Exome Aggregation Consortium (ExAC), Online Mendelian Inheritance in Man (OMIM), Clinical Variant (ClinVar), Human Gene Mutation Database (HGMD), Polymorphism Phenotyping v2 (PolyPhen-2), Sorting Intolerant from Tolerant (SIFT), MutationTaster, Protein Variation Effect Analyzer (PROVEAN), Combined Annotation Dependent Depletion (CADD), Revel, Max Ent Scan, and SpliceAI were used to evaluate the harmful and conservative mutation predictions. Candidate mutations were screened based on clinical phenotypes, and then the pathogenic classification of the candidate mutations was conducted according to the American College of Medical Genetics and Genomics (ACMG) guidelines.

### 2.3 Sanger sequencing

To confirm the *NUS1*:c.279del mutations obtained by whole-exome sequencing, Sanger sequencing was carried out in the proband and his parents. The PCR primers of *NUS1*:c.279del were designed using Primer Premier 6. DNA was extracted using QIAamp DNA Blood Kit (QIAGEN), and the PCR product was sequenced on an ABI 3730xl DNA Analyzer (ABI, USA).

### 2.4 *In silico* protein structure analysis and bioinformatics analysis

The 3D protein structure, including wild-type NUS1 and mutated proteins, was forecasted using SWISS-MODEL (9). The protein–protein interaction (PPI) network related to NUS1 was obtained from STRING (https://string-db.org/), and the biological process analysis of these genes related to NUS1 was carried out.

## 3 Case presentation

### 3.1 Genetic findings

**Figure 1:**
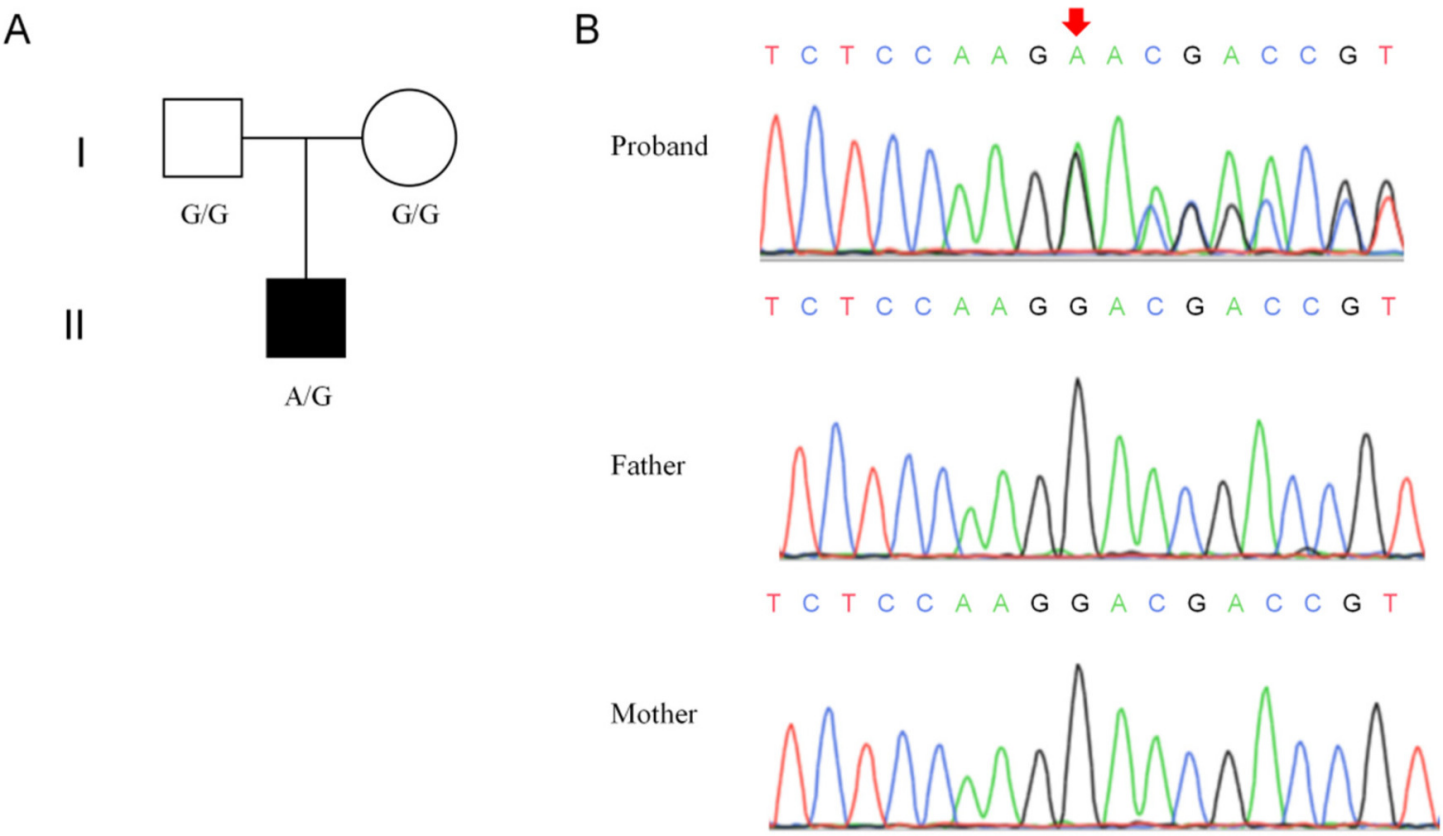
Genetic information of the proband's family. **(A)** Pedigree of the proband's family. **(B)** Results of the Sanger sequencing.

The whole-exome sequencing analysis discovered a novel heterozygous frameshift variant (c.279del, p.L94Wfs*11) in exon 1 of the *NUS1* gene (NM_138459.5) in the proband (Figure 1A). Notably, the c.279del variant of the *NUS1* gene has not been recorded in the gnomAD, 1000 Genomes Project, ESP, ExAC, and HGMD databases. To confirm the whole-exome sequencing results, Sanger sequencing was carried out in the proband and his parents, and the c.279del mutation of the *NUS1* gene was carried by the proband, while no mutation was observed his healthy parents, indicating that the c.279del variant was clarified to be a *de novo* variant in this proband (Figure 1B). Based on the updated ACMG guidelines, the c.279del variant of the *NUS1* gene was classified as pathogenic, and the evidence used was PVS1+PM6+PM2_Supporting.

### 3.2 Evolutionary conservation and *in silico* protein structure analysis

**Figure 2:**
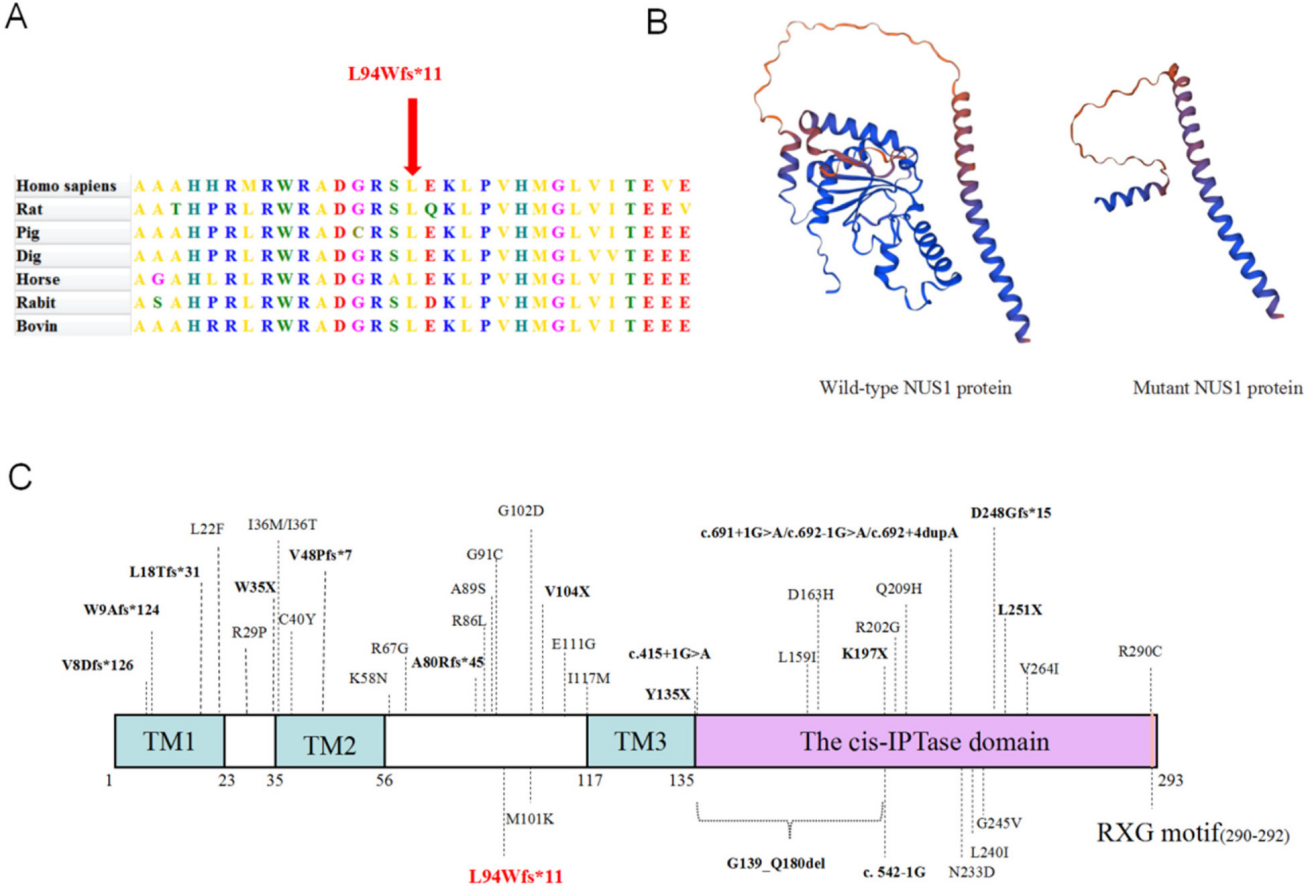
Predicting the impact of the c.279del variation on the NUS1 protein's structure and function. **(A)** Conservation analysis among different species. **(B)** The protein structure of NUS1 is depicted with and without the mutant variations. **(C)** The specific positions of reported variants of the *NUS1* gene and peptide chain schematics.

Multiple sequence alignment of *NUS1* genes in seven species revealed that the amino acids corresponding to the mutations in all species were highly conserved (Figure 2A). SWISS-MODEL analysis revealed that the mutant protein was truncated compared with the wild-type protein (Figure 2B). Furthermore, the c.279del variant of *NUS1* in exon 1 introduces an early terminator, resulting in a truncated protein containing 10 incorrectly encoded amino acids. The truncated protein is composed of 93 correctly encoded amino acids that form part of the transmembrane domain 1 (TM1). However, other functional domains (TM2, TM3, cis-IPTase domain, and RXG motif) are predicted to be removed, leading to a loss of function. In addition, according to previous studies combined with this study, we found 41 *NUS1* variants associated with developmental delay and intellectual disability, including 23 missense variants, 5 nonsense variants, 7 frameshift insertion variants, 5 splicing site mutations, and 1 exon deletion (Figure 2C). Specifically, in Figure 2C, nonsense mutations, frameshift insertions, and splice site mutations are indicated by bold black font, while the mutation identified in this paper is marked with red.

### 3.3 Bioinformatics analysis

**Figure 3:**
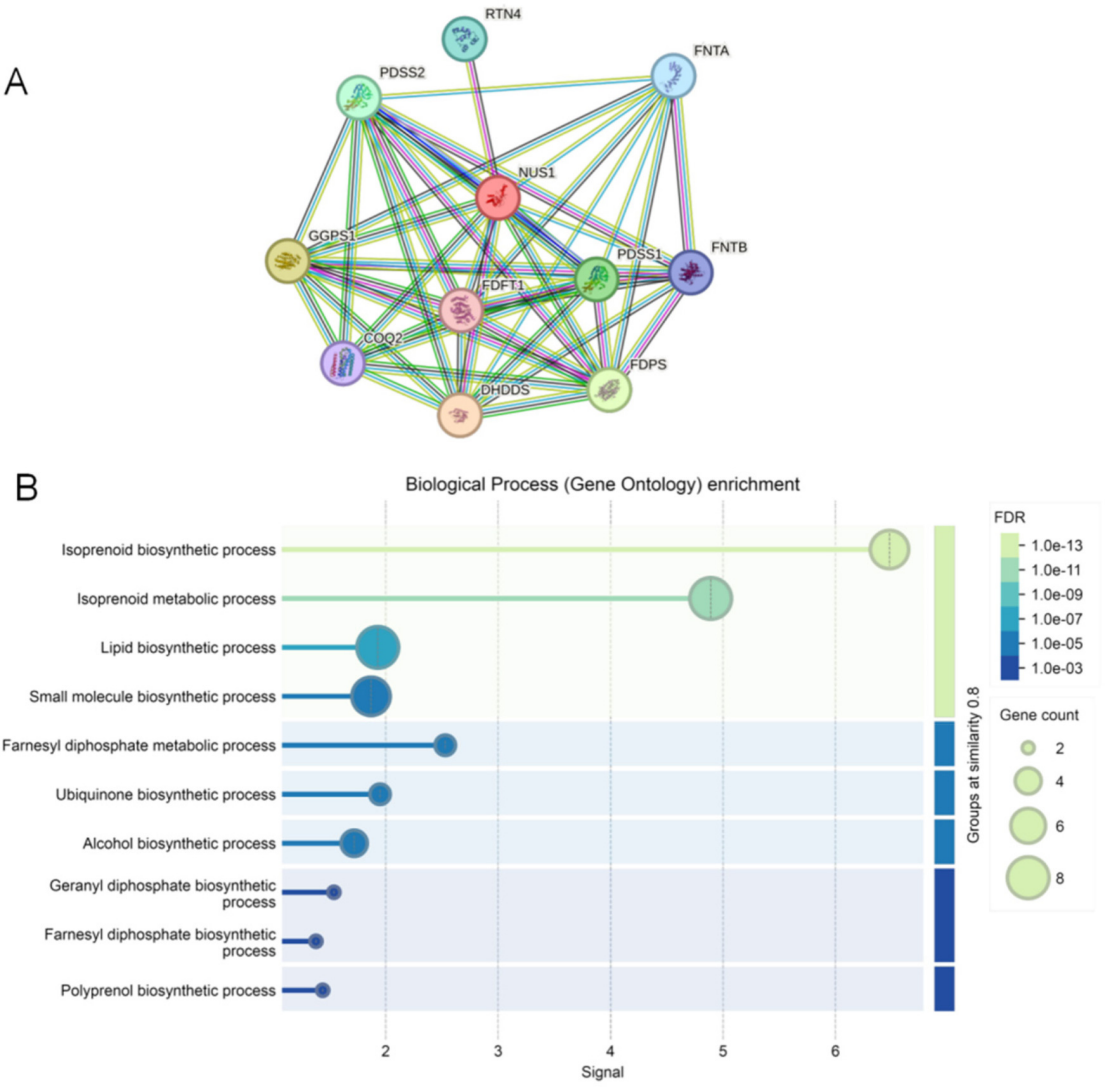
The protein–protein interaction network and GO terms of genes associated with NUS1*.* **(A)** The protein–protein interaction network associated with NUS1. Circles are used to represent nodes, and lines are used to represent edges. **(B)** GO terms of these genes in protein–protein interaction network.

As shown in Figure 3A, the protein–protein interaction network consisted of 11 nodes and 44 edges. These 11 proteins were NUS1 (degree = 10), dehydrodolichyl diphosphate synthase complex subunit (DHDDS) (degree = 9), farnesyl-diphosphate farnesyltransferase 1 (FDFT1) (degree = 9), farnesyl diphosphate synthase (FDPS) (degree = 9), geranylgeranyl diphosphate synthase 1 (GGPS1) (degree = 9), decaprenyl diphosphate synthase subunit 1 (PDSS1) (degree = 9), decaprenyl diphosphate synthase subunit 2 (PDSS2) (degree = 9), farnesyltransferase, CAAX box, subunit alpha (FNTA) (degree = 8), farnesyltransferase, CAAX box, subunit beta (FNTB) (degree = 7), coenzyme Q2, polyprenyltransferase (COQ2) (degree = 7), and reticulon 4 (RTN4) (degree = 1). The biological process analysis found that these genes were enriched in the isoprenoid biosynthetic, isoprenoid metabolic, lipid biosynthetic, and small molecule biosynthetic processes (Figure 3B).

## 4 Discussion

In the current study, we described the genetic diagnosis of a rare *de novo* heterozygous frameshift pathogenic variant in *NUS1* in a boy with developmental delay, intellectual disability, and ASD. This study enlarges the mutation spectrum of *NUS1* and further demonstrates that the mutation of *NUS1* may cause developmental delay and ASD.

The *NUS1* gene, also known as *NGBR* and C6ORF68, consists of five exons, encoding 293 amino acids, and is located on chromosome 6q22.31. *NUS1* encodes the NgBR protein, which serves as a subunit of cis-prenyltransferase. NgBR directly combines with DHDDS to stabilize the dehydrodolichyl diphosphate synthase complex and enhance its enzymatic activity, which is required for protein glycosylation in mammals (4, 5). Therefore, pathogenic NUS1 variants may encode dysfunctional proteins that lack essential functions, disrupting the regulation of protein glycosylation and leading to disease onset. Patients with *NUS1* variants typically exhibit multiple clinical phenotypes, including Parkinson's disease, congenital disorder of glycosylation, MRD55, and others (10–13).

Currently, whole-exome sequencing is the common way to determine the genetic etiology of developmental delay and intellectual disability in children. In this case, a novel heterozygous frameshift variant (c.279del, p.L94Wfs*11) was detected on exon 1 of *NUS1* (NM_138459.5), which was determined to be *de novo* by Sanger sequencing, as the proband's parents do not carry this mutation. Notably, this variant was not reported in the gnomAD, 1000 Genomes Project, ESP, ExAC, and HGMD databases. Bioinformatics analysis predicted that the c.279del mutation of the *NUS1* gene introduced an early terminator leading to protein truncation, resulting in the loss of functional domains, such as TM2, TM3, cis-IPTase domain, and RXG motif, and ultimately leading to the loss of protein function. Following the updated ACMG guidelines, the c.279del (p.L94Wfs*11) variant was classified as pathogenic (PVS1+PM6+PM2_Supporting). In addition, we compared the clinical phenotypes of other reported *NUS1* mutation carriers with the clinical phenotype of the boy reported in this study and discovered that the phenotypes had different degrees of overlap. Clinical presentations of *NUS1* gene mutations typically include seizures, intellectual disability, delayed speech, and delayed movement (2, 13). A recent study demonstrated that patients with *NUS1* variants display movement disorder phenotypes similar to those with DHDDS mutations, prompting the recommendation for *NUS1* gene inclusion in movement disorder diagnostic screening panels (14). The molecular mechanisms underlying NUS1-associated movement disorders remain largely unexplored. Notably, Yu et al. study demonstrated that the NUS1 haploinsufficiency may become a potential pathogenic mechanism for patients with complex movement disorders by inducing lysosomal cholesterol accumulation (15).

ASD currently appears to be a rare phenotypic manifestation among patients with *NUS1* variants, with only a single reported case to date. Hamdan et al. identified a novel *NUS1* variant (c.128_141dup, p.Val48Profs*7) in a boy presenting with developmental delay, intellectual disability, and ASD, representing the first reported association between *NUS1* mutations and ASD (16). Our study similarly identified clinical phenotypes associated with ASD in patients carrying a *de novo NUS1* mutation (c.279del, p.L94Wfs*11), providing further evidence supporting the pathogenic association between *NUS1* variants and ASD. The two *NUS1* mutations associated with ASD were both loss-of-function mutations and located on exon 1. Based on these findings, we speculate that the *NUS1* gene participates in the pathogenic mechanisms underlying ASD and could potentially emerge as a significant focus for ASD studies in the future.

PPI networks play a crucial role in cell function and are essential for understanding biological pathways and their roles in disease development (17). With the rapid development of next-generation sequencing technology and bioinformatics technology, PPI networks have become a powerful technique for elucidating protein–protein interactions in a variety of biological samples (18, 19). Moreover, PPI networks have also proven to be an effective tool for the diagnosis and prevention of certain diseases. To elucidate the pathogenic mechanisms involving NUS1 and its associated genes, we established a PPI network comprising 11 nodes and 44 edges. Subsequently, biological process analysis discovered that these genes were enriched in the isoprenoid biosynthetic, isoprenoid metabolic, lipid biosynthetic, and small molecule biosynthetic processes. Thus, we speculate that *NUS1* and its related genes may have been involved in the MRD55 progression in this case via modulating the above biological processes.

In summary, we performed a genetic etiological analysis of a proband in a Chinese family with MRD55 and ASD, and found a novel heterozygous pathogenic variant (c.279del, p.L94Wfs*11) of the *NUS1* gene, which expanded the phenotype and mutation spectrum of the *NUS1* gene. Furthermore, the identification of the genetic basis of the proband and confirmation of the corresponding loci of his family members will facilitate the genetic counseling of the proband's parents regarding reproduction. Future studies should not only include genotype–phenotype correlation studies, but also involve a functional analysis of pathogenic mechanisms.

## Data Availability

The raw data supporting the conclusions of this article will be made available by the authors, without undue reservation.
